# Prime-boost vaccination with attenuated *Salmonella* Typhimurium *ΔznuABC* and inactivated *Salmonella* Choleraesuis is protective against *Salmonella* Choleraesuis challenge infection in piglets

**DOI:** 10.1186/s12917-017-1202-5

**Published:** 2017-09-11

**Authors:** Giovanni Loris Alborali, Jessica Ruggeri, Michele Pesciaroli, Nicola Martinelli, Barbara Chirullo, Serena Ammendola, Andrea Battistoni, Maria Cristina Ossiprandi, Attilio Corradi, Paolo Pasquali

**Affiliations:** 1 Istituto Zooprofilattico Sperimentale della Lombardia e dell’Emilia Romagna [Experimental Zooprophylactic Institute of Lombardy and Emilia Romagna], 25124 Brescia, Italy; 2 FAO Reference Center for Veterinary Public Health, Department of Veterinary Public Health and Food Safety, Istituto Superiore di Sanità, 00161 Rome, Italy; 3 UCM-UPM, Campus Moncloa, Madrid, Spain; 4 Department of Biology, Università di Roma Tor Vergata, 00133 Rome, Italy; 5 Department of Veterinary Sciences, University of Parma, Parma, Italy

**Keywords:** Attenuated and inactivated vaccines, Mucosal vaccine, *Salmonella* Choleraesuis, Piglets, Salmonellosis, IFN-gamma

## Abstract

**Background:**

*Salmonella enterica* serovar Choleraesuis (*S*. Choleraesuis) infection causes a systemic disease in pigs. Vaccination could represent a solution to reduce prevalence in farms. In this study, we aimed to assess the efficacy of an attenuated strain of *Salmonella enterica* serovar Typhimurium (*S*. Typhimurium *ΔznuABC*) against *S*. Choleraesuis infection. The vaccination protocol combined priming with attenuated *S*. Typhimurium *ΔznuABC* vaccine and boost with an inactivated *S*. Choleraesuis vaccine and we compared the protection conferred to that induced by an inactivated *S*. Choleraesuis vaccine.

**Methods:**

The first group of piglets was orally vaccinated with *S.* Typhimurium *ΔznuABC* and boosted with inactivated *S*. Choleraesuis, the second one was intramuscularly vaccinated with *S.* Choleraesuis inactivated vaccine and the third group of piglets was unvaccinated. All groups of animals were challenged with a virulent *S.* Choleraesuis strain at day 35 post vaccination.

**Results:**

The results showed that the vaccination protocol, priming with *S.* Typhimurium *ΔznuABC* and boosted with inactivated *S*. Choleraesuis, applied to group A was able to limit weight loss, fever and organs colonization, arising from infection with virulent *S*. Choleraesuis, more effectively, than the prime-boost vaccination with homologous *S*. Choleraesuis inactivated vaccine (group B).

**Conclusion:**

In conclusion, these research findings extend the validity of attenuated *S.* Typhimurium *ΔznuABC* strain as a useful mucosal vaccine against *S.* Typhimurium and *S*. Choleraesuis pig infection. The development of combined vaccination protocols can have a diffuse administration in field conditions because animals are generally infected with different concomitant serovars.

## Background

*Salmonella enterica* serovar Typhimurium (*S.* Typhimurium) and *Salmonella enterica* serovar Choleraesuis (*S*. Choleraesuis) are the main etiological agents of salmonellosis in pigs. The former is responsible for enterocolitis in pigs [1] and zoonotic infections through consumption of contaminated pork products [2]. Conversely, *S*. Choleraesuis induces septicemia, pneumonia, enterocolitis, hepatitis, meningo-encephalitis and abortion in pigs [3], causing significant economic losses in pig industries [1, 4].

The principal tools for controlling typhoid-*Salmonella* are antimicrobials, vaccines, farm management and biosecurity plans. Differently, clinical cases of non-typhoid *Salmonella* are rare and, generally, pigs are sub-clinically infected [5, 6]. Recent monitoring programs had demonstrated that number of *Salmonella* multi-drug-resistant strains (typhoid and non-typhoid) has been incrementing. Antimicrobials are generally used for metaphylaxis or treatment of enterocolitis or other diseases caused by several pathogens in post-weaning phase. This activity has also determined an increment of multidrug strains frequently present in herds without clinical evidence (included *Salmonella* strains). It is also well recognized the ability of these bacteria to acquire drug-resistance from other bacteria by plasmid-transfer. However, due to the diffusion of multi-drug-resistant strains, the further uses of antibiotics is highly discouraged [7]. In alternative, vaccines could be an efficacious solution in reducing shedding of *Salmonella* spp., especially when vaccination is combined with biosecurity strategies [8, 9]. Unfortunately, safe and immunogenic live *Salmonella* attenuated vaccines are limited on the market and their efficacy against heterologous challenge is still not completely known [6, 10].

In recent years we have shown that mutant strains of *S.* Typhimurium, deleted of z*nuABC* genes (*S.* Typhimurium Δ*znuABC*) are drastically attenuated and have significant potential as live vaccine [11]. In fact, we have demonstrated that this strain is attenuated and protective in both mouse and pig models of infections, against challenges with *Salmonella* Typhimurium wild type [12–16].

The aim of this study was to assess whether *S.* Typhimurium Δ*znuABC*, boosted with the inactivated *S*. Choleraesuis, is able to exert protective effects versus wild type *S*. Choleraesuis and to compare its efficacy related to that induced by an inactivated *S*. Choleraesuis prime-boost vaccine. We boosted an attenuated *S.* Typhimurium vaccine with an inactivated *S*. Choleraesuis because attenuated vaccines favor T-cell response, which is very highly protective against intracellular bacteria as *Salmonella.* However, it is only partially protective against a typhoid *S.* Choleraesuis infection. For this reason, we boosted with a homologous inactivated vaccine, favoring maturation of affinity of the immune response and, in particular, of B cell, to complete immune response. In this way, antibodies versus all epitopes of this strain were generated, ensuring protection against extracellular bacteria which systemically spread during typhoid-infection.

## Methods

### *Salmonella* spp. cultures

As a vaccine strain, we used an attenuated *S.* Typhimurium *ΔznuABC* strain containing a cassette for chloramphenicol resistance inserted in the *znuABC* locus made resistant also to streptomycin by P-22 mediated transduction of *hsdR::spec* allele. Briefly, a fragment containing chloramphenicol resistance cassette was amplified from plasmid pKD3 and electroporated in a *S*. Typhimurium virulent strain (ATCC 14028). Correct insertion was confirmed by inability to grow in a zinc-poor medium and by PCR. Phage P-22 was used to transduce the mutation into a *S*. Typhimurium strain also resistance for streptomycin (transduction of *hsdR::spec* allele) [11].

*S*. Choleraesuis was cultivated in BHI broth (Reparto produzione Terreni, IZSLER, Brescia) at 37 °C overnight in a fermenter (Sartorius, Biostat Cplus, Italy) and then was inactivated with formaldehyde 0.8% (*v*/v). The final concentration of 2 × 10^10^ CFU/ml was determined by a Cell Density Meter (WPA CO 8000 Biowave Cell Density Meter, Biochrom Ltd., Cambridge, UK) and then absorbed in 10 mg/ml of Aluminum hydroxide. Complete inactivation was confirmed by cultivating an aliquot of prepared vaccine in BHI agar at 37 °C for 48 h. (Vaccine and Reagent Preparation Laboratory of the “Experimental Zooprophylactic Institute of Lombardy and Emilia Romagna”, IZSLER).

*S.* Typhimurium *ΔznuABC* and virulent *S.* Choleraesuis were grown overnight at 37 °C in Brain Heart Infusion (Oxoid Ltd., UK), harvested by centrifugation and then washed twice in ice-cold phosphate buffer solution (PBS) (Sigma–Aldrich, Italy).

### Experimental design

Experiments were authorized by the national authorities in accordance with Italian and European regulations (D.lgs 116/1992 implementing the European directive n° 86/609/CEE) and were conducted under the supervision of certified veterinarians.

Eighteen weaned piglets (28 days-old) were housed in the animal facility of the IZSLER, acclimatized for one week before the experiment and checked to be *Salmonella*-free using microbiological and serological analysis. The health-status of the native farm was monitored during last years. Particularly, sows were negative because they did not produce antibodies against a broad range of serogroups (IDEXX Herd-check Swine Salmonella Antibody test kit) and fecal samples, collected during last weeks of pregnancy, were negative for *Salmonella* culture performed in accordance with ISO 6579:2002. Similarly, serum and feces of their piglets, enrolled in this study, were collected a week before movement to test seroconversion and presence of positive culture. Piglets were divided into three groups (6 animals per group).

Vaccines were administered a week after their arrival (35 days-old) following a protocol described below. Group A, vaccinated by oral gavage, with 5 × 10^7^ CFU of *S.* Typhimurium *ΔznuABC* dissolved in 20 ml of sodium bicarbonate buffer, and boosted after two weeks with an intramuscular administration of inactivated *S.* Choleraesuis at the dose of 2 × 10^9^ CFU/ml. Group B was intramuscularly vaccinated with *S.* Choleraesuis inactivated vaccine and boosted after two weeks at the dose of 2 × 10^9^ CFU/ml. Group C was maintained as an unvaccinated naïve control. Fecal samples were collected at 1, 5, 13, 18 and 33 days after vaccination to determine the amount of *S.* Typhimurium *ΔznuABC* attenuated strain.

All groups were challenged, by gavage, with 5 × 10^8^ CFU of *S.* Choleraesuis virulent strain dissolved in 20 ml of sodium bicarbonate buffer, at day 35 from first vaccination. Temperature was measured at 3, 4, 5 and 7 days after infection. Animals were weighed at first vaccination and before necropsy (day 47 from first vaccination). Tonsils, ileocecal lymph nodes, spleen, liver, intestinal content of ileum, cecum, colon and jejunum were collected during necropsy for microbiological analyses and gross lesions of organs were recorded.

### Microbiology

Fecal shedding and organ colonization of *S.* Choleraesuis and *S.* Typhimurium *ΔznuABC* were determined using the ISO 6579: 2002/ Amendment 1: 2007 protocol. Samples were weighed and homogenized in 9 parts of Buffered Peptone Water (BPW) (Oxoid Ltd., UK). This solution was first used to perform a Serial Dilution in BPW. All BPW samples (diluted or not) were incubated at 37 °C for 18 ± 3 h. Afterwards, 0.1 ml of BPW cultures were seeded on modified semisolid Rappaport-Vassiliadis agar (MSRV) plates (Oxoid Ltd., UK) and incubated at 41.5 °C for 48 h for selection and enrichment of *Salmonella.* A loopful of culture from an MSRV plate was streaked onto Xylose-Lysine-Deoxycholate Agar (Oxoid Ltd., UK) and Brillant Green Agar (Oxoid Ltd., UK) plates and then incubated at 37 °C overnight. Suspect *Salmonella* colonies were subjected to biochemical identification by BBL Enterotube II (BD Franklin Lakes, NJ USA) and serological identification using *Salmonella* group-specific antisera. XLD agar allows a primary distinction of H_2_S+ and H_2_S- *Salmonella* strains. This is a semi-quantitative approach consisting in application of the qualitative approach to each ten-fold dilution.

The semi-quantitative approach allows us to establish a range of concentration of *Salmonella* in a sample. This method limits enumeration of *Salmonella* in a sample, but it reduces presence of concomitant bacteria and favors isolation of *Salmonella*. Results express a range of concentration in each sample as reported in Table 1.

**Table 1:** Semi-quantitative approach for count of *Salmonella*-colonies

Ten-fold dilution positive	results express in	
	LOG_10_	CFU/g
Negative	0	Negative also after enrichment
1:10^1^	1	1–10 CFU/g
1:10^2^	2	11–100 CFU/g
1: 10^3^	3	101–1000 CFU/g
1: 10^4^	4	1001–10,000 CFU/g
1: 10^5^	5	10,001–100,000 CFU/g
1: 10^6^	6	100,001–1,000,000 CFU/g
1: 10^7^	7	1,000,001–10,000,000 CFU/g

Results of positive ten-fold dilution are expressed as LOG_10_ and correspond to a range of CFU/g. They represent a semi-quantitative approach to establish concentration in a sample

### Interferon-γ production

At necropsy, ileocecal lymph nodes, draining the site of inoculum, were collected from animals of groups A-C to compare IFN-γ concentration after challenge. Lymph nodes were homogenized by a mortar in fetal calf serum (Gibco Life Technologies, Paisley, UK) + 5% DMSO (Sigma-Aldrich, St.Louis, MO, USA) and filtered with gauze to retain coarse particles. An aliquot of cell suspension was then stored at −80 °C using a proteinase inhibitor (Protease Inhibitor Cocktail kit, Thermo Scientific, Rockford, IL., USA), until use. IFN-γ production was assessed by a sandwich ELISA (Pig Interferon-γ; −IFN-γ, ELISA Kit, Cusabio, P.R. China), in accordance with the manufacturer’s instructions. The exact amount of IFN-γ production was then calculated by normalizing the result using total protein content. Total protein content was determined by application of Lambert-Beer Law [17]. In particular, The amount of the tissues after homogenization was standardized by total protein concentration and a specific volume of each homogenate was analyzed by ELISA kit. In conclusion, results were determined by the concentration of cytokine per ng of total protein (pg of IFN-g/ng of total protein).

### Serology

The serological examination was performed using a commercial indirect ELISA test capable of detecting antibodies against *Salmonella* serogroups B, C1 and D (Herd-Check Swine Salmonella Antibody Test Kit, IDEXX Laboratories Inc., Switzerland). The test was carried out in accordance with the manufacturer’s instructions and analyzed at an optical density of 450 nm. Results were expressed as a sample to positive ratio [S:P ratio = (OD of sample – OD of negative control)/(OD of positive control – OD of negative control)].

### Statistical analysis

All statistical analyses were performed using GraphPad Prism (vers. 4.0) software (GraphPad Inc., San Diego, CA, USA). Data related to temperature and antibody titers were analyzed using Two-Way ANOVA and completed with the Bonferroni post-test. Data related to organ colonization were analyzed using the Kruskal–Wallis test (non-parametric one-way analysis of variance – ANOVA) and completed with the Dunn’s Multiple Comparison post-test. A *P*-value <0.05 was considered to indicate statistically significant differences.

## Results

### *S*. Typhimurium *ΔznuABC* strain is not detectable in feces 18 days after vaccination

**Fig. 1:**
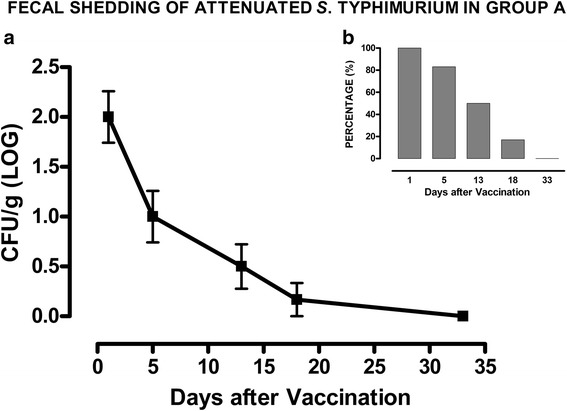
Attenuated *S*. Typhimurium *ΔznuABC* is not detectable from day 18 after vaccination. In **a**, symbols depict the mean concentration of *S*. Typhimurium *ΔznuABC* (expressed as LOG_10_ CFU/g) in group A at different time points (day 1, 5, 13, 18, 33 after vaccination). Bars represent the standard deviation. In **b**, columns represent the percentage of shedder piglets of group A at different time-points. *S*. Typhimurium *ΔznuABC* is not detectable from day 18 after vaccination. Group B and C are not shown because negative. Depicted microbiological results derived from the semi-quantitative approach

Firstly, we wanted to reconfirm the safety and the limited environmental contamination of attenuated *S.* Typhimurium *ΔznuABC.* For this reason, fecal samples were weekly collected after vaccination in animals of group A, to estimate the amount of *S*. Typhimurium *ΔznuABC* (Fig. 1). Fecal samples were also collected in animals of groups B and C, but these animals did not shed *Salmonella* spp. (data not shown). The attenuated strain was shed up to 18 days after vaccination and the number of shedder pigs and the concentration of bacteria sharply decreased from vaccination and thereafter.

### Combined vaccination protocol reduces clinical symptoms induced by *S*. Choleraesuis infection

We analyzed the efficacy of vaccination, considering the clinical, microbiological and immunological parameters influenced by a *S.* Choleraesuis infection.

**Fig. 2:**
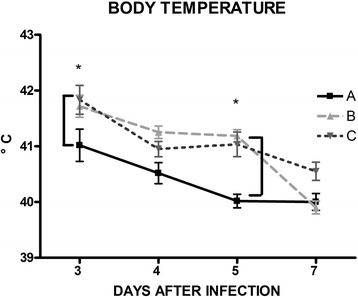
Vaccination with attenuated *S.* Typhimurium *ΔznuABC* prevents fever*.* Body temperature of groups A, B and C is shown at different time points (day 3, 4, 5 and 7 after challenge). Symbols represent mean and bars standard deviation. Symbols (*) represent differences statistically significant among groups with *p* < 0.01

The mean temperatures of the three groups are shown in Fig. 2. Overall, irrespective to the treatment, piglets challenged with the wild-type strain of *Salmonella* Choleraesuis showed a raise of body temperature which tend to drop back down to baseline one week after infection.

Nevertheless, we observed that the raise of body temperature of group B and C was higher than that of group A, especially at 3 and 5 days after infection (*p* < 0.05).

**Fig. 3:**
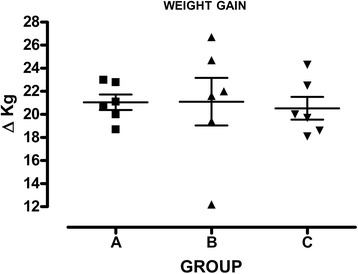
Attenuated strain of *S.* Typhimurium does not retard the weight gain of piglets*.* Symbols represent the weight gain of eighteen animals, divided into groups A, B and C, while vertical bars represent the standard deviation from the beginning to the end of the study (day 0 and 47). No differences are recorded. The weight of animals vaccinated with the attenuated strain of *S.* Typhimurium is not different from other groups

We further compared the weight gain from vaccination to the killing to assess if different protocols of vaccination could influence the weight gain. As depicted in Fig. 3, we did not record any difference among groups throughout the observation in terms of weight gain.

### Combined vaccination protocol significantly reduces organ colonization after challenge infection with virulent *S*. Choleraesuis

**Fig. 4:**
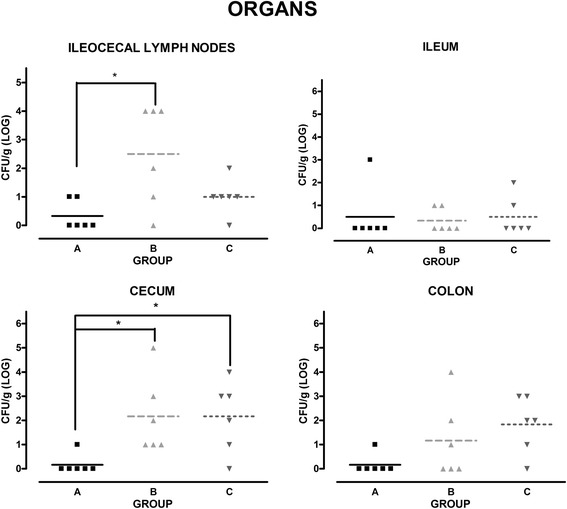
*S*. Typhimurium *ΔznuABC* vaccine reduces organ colonization. Amount of *S*. Choleraesuis in lymph nodes, ileum, cecum and colon of group A-C piglets was determined 12 days after challenge. Depicted microbiological results derived from the semi-quantitative approach. Each symbol represents microbiological results obtained from each animal and each bar represents mean concentration. Differences are statistically significant in cecum between group A and the other groups (*p* < 0.05) and in lymph nodes between group A and B

Organ colonization tested at 12 days after challenge was low and barely detectable in many organs. Tonsils, spleen, liver and intestinal content of jejunum were colonized only in a very limited percentage (data not shown). Organs which showed the most reliable colonization were cecum and ileocecal lymph nodes (Fig. 4). Overall, we observed that piglets of group A, treated with combined vaccination protocol (oral administration of attenuated *S.* Typhimurium *ΔznuABC* vaccine boosted after two weeks with an intramuscular injection of inactivated *S.* Choleraesuis), showed a reduction of *Salmonella* Choleraesuis colonization and that inactivated vaccine, administered to piglets of group B, did not exert an analogous effect. In particular, cecum colonization of group A was statistically different from group B and C, and ileocecal lymph nodes colonization of group A was significantly lower than group B.

### Combined vaccination protocol induces both innate and humoral immunity in response to a *S*. Choleraesuis challenge infection

**Fig. 5:**
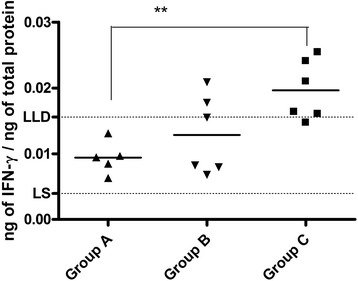
*S*. Choleraesuis challenge infection induces IFN-ɤ production. Symbols and bars represent piglets and IFN-ɣ mean concentration, respectively. Concentration of IFN-ɤ was normalized in relation to total protein content. Difference was statistically significant between the unvaccinated group (C) and group A vaccinated with the attenuated strain ** (p < 0.05). LLD indicates Lower Limit of Detection (15.6 pg/ml); LS indicates Limit of Sensitivity (minimum detectable dose, 3.9 pg/ml)

We estimated the response induced by vaccination and infection, by analyzing IFN-γ and humoral response. At day 47 after the first vaccination (i.e. 12 days after the challenge infection), IFN-γ was produced in a higher concentration in the ileocecal lymph nodes from piglets of group C compared to those from piglets of vaccinated groups (Fig. 5). In particular, IFN-γ concentration was approximately 0.02 ng/ml in group C and the difference was statistically significant when compared to the concentration in group A. On the contrary, the concentration of IFN-γ in leukocytes of group B was not statistically different from that in group C.

**Fig. 6:**
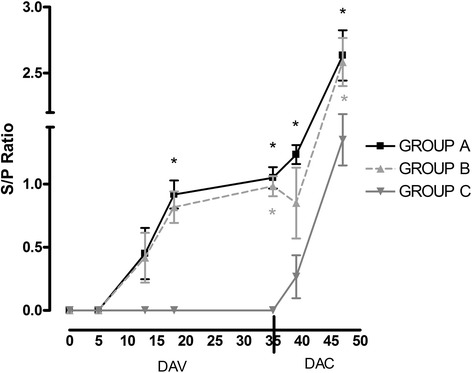
Attenuated *S.* Typhimurium *ΔznuABC* vaccine and inactivated *S*. Choleraesuis vaccine induce antibody production. Symbols and bars represent mean and standard deviation of s/p ratio in 3 groups, respectively. The pattern of humoral immunity is similar between group A (vaccinated with Attenuated *S*. Typhimurium *ΔznuABC*) and in group B (vaccinated with killed *S*. Choleraesuis). The X-axis is divided into two segments to differentiate between antibody response after vaccination (DAV) and challenge (DAC). Differences are statistically significant between vaccinated piglets with attenuated strain and unvaccinated piglets from day 18 after vaccination. On the contrary, differences are statistically different between vaccinated piglets with inactivated strain and unvaccinated piglets only at day 35 and 47 after vaccination. Gray star indicates significant difference between groups B and C, while black star indicates significant difference between group A and C

Antibody response was monitored after vaccination and after challenge in all groups of piglets enrolled in this study. Vaccinated piglets of group A and B had a humoral response starting from the first week after vaccination, with an increasing of s/p ratio (OD) thereafter; in group C, on the other hand, the response started after infection. Humoral responses were similar between the two vaccinated groups and the differences observed were statistically significant when comparing the group A to C (Fig. 6).

## Discussion

Salmonellosis is a public health problem primarily caused by consumption of pork products contaminated with *S*. Typhimurium [2]. On the contrary, *S*. Choleraesuis, a host-adapted serovar in pigs, causes a typhoid-like disease in piglets, which is characterized by reduced growth and, in the most severe cases, a high mortality rate, and hence mainly representing an economic problem [1, 18]. *S*. Choleraesuis is not considered to be a major agent of zoonotic infections, although some cases of human infection have been recorded, especially in Asia [8].

Vaccination of pigs could represent a valid control system in countries with a high prevalence of salmonellosis in animals. Attenuated vaccines are more effective than inactivated ones in protecting against enteric diseases, due to their ability to induce cell-mediated and mucosal immunity [19]. To address this issue, in previous study we assessed the safety and efficacy of *S*. Typhimurium *ΔznuABC* strain in different models of infection [11–16]. In the current work, an attenuated *S*. Typhimurium *ΔznuABC* boosted with an inactivated *S*. Choleraesuis vaccine was compared to an inactivated *S*. Choleraesuis vaccine in providing immune-based protection against a *S*. Choleraesuis challenge infection.

The cross-protection had already been investigated in a mouse challenge infection [12], suggesting that *S*. Typhimurium *ΔznuABC* is able to induce partial protection against heterologous challenge with *S*. Choleraesuis. We therefore set up a new vaccination protocol based on an oral vaccination with attenuated *S*. Typhimurium *ΔznuABC* followed by an intramuscular boost with an inactivated *S*. Choleraesuis vaccine after two weeks.

As control groups, piglets were vaccinated and boosted with inactivated *S.* Choleraesuis vaccine or were kept as naïve unvaccinated ones. Attenuated vaccines induce a more effective T-cell involvement against facultative intracellular bacteria, such as *Salmonella* [20, 21]. We hypothesized that, a boost with an inactivated vaccine may favor the maturation of affinity of the immune response and the production of mucosal and serum antibodies against somatic antigens, thus completing the host immune response and enhancing the efficacy of the attenuated strain. The shedding pattern of *S.* Typhimurium *ΔznuABC* was characterized by a sharp decline within few days and then it was not detectable in feces after five weeks. Those data corroborate findings and data previously published that showed a limited and self-limiting persistence of *S*. Typhimurium *ΔznuABC* [14–16]. We found that, when challenged with virulent *S.* Choleraesuis, piglets vaccinated with the prime-boost protocol with attenuated *S*. Typhimurium *ΔznuABC* and inactivated *S*. Choleraesuis vaccine showed a lower increase in body temperature compared to the other groups. In this study, we observed a modest colonization of organs which suggests that the employed *Salmonella* Choleraesuis strain is not highly virulent and/or that it needs more time or different condition to develop the acute form. Nonetheless, cecum colonization of group A was lower than colonization of groups B and C, whilst ileocecal lymph nodes colonization was lower only in comparison to group B.

These findings suggest that this combined vaccination protocol is able to exert protection, while prime-boost vaccination with inactivated *S*. Choleraesuis vaccine does not curb the progression of infection and organ colonization. These data support the hypothesis that vaccination with the attenuated *S*. Typhimurium *ΔznuABC*, previously investigated as safe and effective against *S*.Typhimurium [14–16], followed by boost with inactivated *S*. Choleraesuis vaccine could decrease the number of pigs carrying different serovars of *Salmonella* in field conditions.

Studies focused on the heterologous protection have previously been published. Schwarz et al. [22] demonstrated that an attenuated strain of *S*. Choleraesuis reduced the prevalence of *Salmonella* in carrier pigs at the slaughterhouse. This attenuated strain was used on a farm infected with *S*. Brandenburg, *S*.Typhimurium and *S*. Agona. Moreover, cross-protection was also documented between host-specific strains. House et al. [23] demonstrated that an attenuated *S*. Choleraesuis vaccine, licensed for swine, was more efficacious than an autogenous *Salmonella* bacterin in pregnant cows infected with *S*. Montevideo. We can infer that there is an overlap between antigenic determinants that induce protection. Other studies, however, should be performed to identify the common virulence factors of different serovars involved in animal salmonellosis. This knowledge is necessary to develop an efficacious multivalent *Salmonella* vaccine.

To better understand the protection induced by vaccination, we analyzed host response after challenge, considering IFN-γ and antibody production. IFN-γ was chosen as a paradigmatic cytokine for a Th1 cell mediated immune response which is known to be involved in the control of *Salmonella* infection. Particularly, IFN-γ is an important cytokine produced by natural killer cells (NK) and T-cells, in response to phagocytosis of *Salmonella* by macrophages and other antigen presenting cells (APC) during the earlier phase of its systemic dissemination [19, 24]. In our study, the concentration of IFN-γ in groups A and B was lower than that of group C, at day 12 after infection suggesting that IFN-γ production is a marker of the host response, as reported previously [15]. Moreover, we obtained a seroconversion of piglets one week after vaccination that significantly increase after challenge with virulent *S.* Choleraesuis. These results are in line with the study of Husa et al. [25] that compared the safety, cross-protection and serological response of two commercial live *S.* Choleraesuis in response to experimental challenge infection with *S.* Typhimurium. *S.* Choleraesuis vaccines, indeed, induced a humoral response characterized by an increase in antibody titers after vaccination, which rapidly rose after challenge with the heterologous strain.

## Conclusion

In conclusion, we produce scientific evidence that a vaccination protocol, characterized by combination of attenuated and inactivated vaccines of *S*. Typhimurium and *S*. Choleraesuis, is effective against challenge infection with *S.* Choleraesuis. In perspective, these data suggest that it is could be possible to develop new effective vaccine strategies for the treatment of animals simultaneously infected by different serovar of *Salmonella*, a condition that commonly occurs in field conditions.

## Abbreviations

ANOVA: Analysis of variance
BPW: Buffered peptone water
CFU: Colony Forming Unit
DMSO: DiMethyl sulfoxide
IFN-γ: Interferon-gamma
IZSLER: Istituto Zooprofilattico Sperimentale della Lombardia e dell’Emilia Romagna [Experimental Zooprophylactic Institute of Lombardy and Emilia Romagna]
ml: milliliter
MSRV: Modified semisolid rappaport vassiliadis agar
ng: nanogram
NK: Natural killer cells
OD: Optical density
PBS: Phosphate buffer solution
XLD: Xylose-lysine-deoxycholate agar
